# The Influence of Estrogens on the Biological and Therapeutic Actions of Growth Hormone in the Liver

**DOI:** 10.3390/ph5070758

**Published:** 2012-07-19

**Authors:** Mercedes de Mirecki-Garrido, Borja Guerra, Carlos Mateos-Díaz, Roberto Jiménez-Monzón, Nicolás Díaz-Chico, Juan C. Díaz-Chico, Leandro Fernández-Pérez

**Affiliations:** Oncology-Molecular and Translational Endocrinology Group, Clinical Sciences Department, Faculty of Health Sciences, Associate Unit of University of Las Palmas de Gran Canaria and Biomedical Institute “Alberto Sols”-CSIC, Las Palmas de G.C. 35016, Spain

**Keywords:** growth hormone, 17β-estradiol, liver, growth, metabolism, STAT5

## Abstract

GH is main regulator of body growth and composition, somatic development, intermediate metabolism and gender-dependent dimorphism in mammals. The liver is a direct target of estrogens because it expresses estrogen receptors which are connected with development, lipid metabolism and insulin sensitivity, hepatic carcinogenesis, protection from drug-induced toxicity and fertility. In addition, estrogens can modulate GH actions in liver by acting centrally, regulating pituitary GH secretion, and, peripherally, by modulating GHR-JAK2-STAT5 signalling pathway. Therefore, the interactions of estrogens with GH actions in liver are biologically and clinically relevant because disruption of GH signaling may cause alterations of its endocrine, metabolic, and gender differentiated functions and it could be linked to dramatic impact in liver physiology during development as well as in adulthood. Finally, the interplay of estrogens with GH is relevant because physiological roles these hormones have in human, and the widespread exposition of estrogen or estrogen-related compounds in human. This review highlights the importance of these hormones in liver physiology as well as how estrogens modulate GH actions in liver which will help to improve the clinical use of these hormones.

## 1. Introduction

The liver responds in a sex-specific manner to growth hormone (GH) and sex hormones. GH is the main regulator of body growth, somatic development, metabolism, sex-differentiated functions in the liver, and aging [1,2,3,4,5,6,7]. Because the liver has the highest levels of GH receptor (GHR), it is a major target for GH; however, virtually all human tissues are responsive to GH. The sex-specific GH secretion from pituitary has been shown to have a great impact on hepatic transcriptional regulation [2,4,8,9]. The Signal Transducer and Activator of Transcription (STAT)-5b is of particular importance in the regulation of the endocrine, metabolic, and sex-differentiated actions of GH in the liver. In the liver, GHR-STAT5 signaling regulates the expression of the target genes that are associated with several physiological processes, such as body growth, the cell cycle, and lipid, bile acid, steroid, and drug metabolism. Importantly, the disruption of GHR-JAK2-STAT5 signaling is associated with liver disease, which includes fatty liver, fibrosis, and hepatocellular carcinoma.

A major natural estrogen in mammals, 17β-estradiol (E2) has physiological actions that are not limited to male or female reproductive organs [10,11]. Estrogens exert their physiological influence through two estrogen receptor (ER) subtypes, ERα and ERβ. These subtypes belong to the nuclear receptor family of ligand-activated transcription factors [12]. Together with a mechanism based in ligand-activated transcription, estrogens can modulate gene expression by using a second mechanism in which the ERs interact with other transcription factors through a process referred to as transcription factor crosstalk. Estrogen may also elicit effects through non-genomic mechanisms, which involve the activation of protein kinase cascades via membrane-localized ERs. Moreover, the mechanisms involved in ER signaling are influenced by cell phenotype, the target gene, and activity or crosstalk with other signaling networks.

The potential interactions between estrogens and the GH-regulated endocrine, metabolic and sex-differentiated functions in the liver are biologically and clinically relevant. Estrogens can modulate GH actions in the liver by acting centrally to regulate pituitary GH secretion and modulating GH signaling peripherally. Most previous studies have focused on the influence of estrogens on pituitary GH secretion [13]; however, there is also strong evidence that estrogens modulate GH action at the level of GHR expression and signaling. In particular, E2 has been shown to induce suppressor of cytokine signaling (SOCS)-2 and -3, which are protein inhibitors for cytokine signaling that in turn negatively regulate the GHR-JAK2-STAT5 pathway [11,14,15,16,17,18,19]. Finally, the liver is a direct estrogen target because it expresses ERα [12], which is connected to liver development [20], the regulation of hepatic metabolic pathways [11], growth [21], protection from drug-induced toxicity [22], hepato-carcinogenesis [23], fertility [24], lipid metabolism and insulin sensitivity [11,25].

Estrogen-GH interplay is clinically relevant because of the physiological roles that these hormones have in mammals and the widespread use of estrogen and estrogen-related compounds in humans. This relevance has been supported by clinical observations in which the administration of pharmacological estrogen doses in humans impairs the GH-regulated endocrine and metabolic functions in the liver [26]. Thus, the deficiency of GH or E2 activities and the interaction of estrogen with GH biology may dramatically influence liver physiology during development and in adulthood. This review highlights the importance of these hormones in liver physiology and describes how estrogens can modulate GH action in the liver. A better understanding of estrogen-GH interplay will lead to improved clinical management of these hormones.

## 2. Physiological Basis of Pituitary GH Secretion

**Figure 1:**
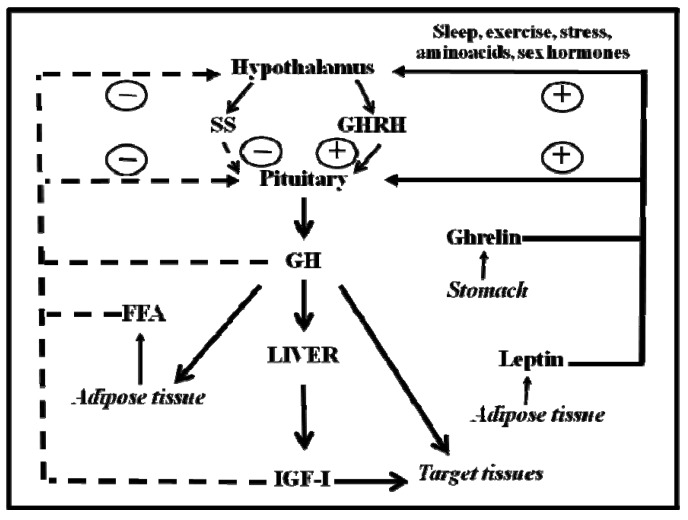
Schematic representation of the somatotropic axis. GHRH and SS, two hypothalamic hormones, control the synthesis and release of GH from the pituitary gland. GHRH is negatively (dashed lines) regulated by feedback from blood GH and IGF-I concentrations. FFA inhibits GH release, whereas leptin and ghrelin stimulate it. Sex hormones and other factors act centrally to stimulate the release of GH. Circulating GH directly stimulates IGF-I production in many organs. IGF-I production in the liver provides the main source of blood IGF-I. GH directly affects many target tissues, sometimes independent of the IGF-I action.

GH is a polypeptide that is secreted primarily from the somatotrophs within the anterior pituitary gland. In addition to the pituitary gland, GH is produced in extra-pituitary tissue, which indicates that GH has local paracrine-autocrine effects that are distinct from its classic endocrine-somatotropic effects [27]. The regulation of pituitary GH secretion involves a complex neuroendocrine control system that includes the participation of several neurotransmitters and the feedback of hormonal and peripheral (metabolic) factors [28]. Figure 1 shows that GH secretion from the pituitary gland is regulated by two major hypothalamic peptides: GH-releasing hormone (GHRH) and the inhibitory hormone somatostatin (SS). The balance of these stimulating and inhibiting peptides is indirectly affected by many physiological stimulators (e.g., nutrients, sleep, exercise, thyroid hormones and sex hormones) and inhibitors (e.g., glucocorticoids, Insulin-like Growth Factor (IGF)-I, GH). The final integration of these signals occurs in the hypothalamus. Pituitary GH secretion is reduced mainly by the negative feedback of two circulating signals: the pituitary GH itself and the liver-derived IGF-I, which is produced by the GH. In addition to hypothalamic (GHRS, SS) and endocrine (IGF-I, GH) factors, other peripheral (metabolic) factors, such as insulin, glucose, amino acids, free fatty acids (FFA), leptin, neuropeptide Y, and ghrelin, influence pituitary GH release. These factors, which appear to coordinate the metabolic status of the organism with GH secretion, are primarily related to or derived from the metabolic status of the organism; this relationship is consistent with the GH role in regulating substrate metabolism, adiposity, and growth. This role is exemplified by adiposity, which is a powerful negative regulator of GH secretion. FFA can act directly on the pituitary gland to inhibit the GH release, which is postulated to complete a feedback loop because GH stimulates lipid mobilization. In addition, adipocytes produce the hormone leptin, which, in contrast to FFA, stimulates GH secretion in rodents at the hypothalamus level [29]. Finally, ghrelin is another GH-secretory factor that is highly expressed in the endocrine cells of the stomach [30]. On the other hand, the selective lack of ghrelin receptor signaling in humans may lead to a syndrome characterized by short stature [31], and ghrelin analogs have been shown to effectively enhance serum IGF-I levels in humans [32].

Sex steroids are also physiological regulators of pituitary GH secretion and regulate sex-specific liver physiology. Both neonatal and post-pubertal sex steroids control the ability of the hypothalamus to drive the sexual dimorphism of pituitary GH secretion in adulthood [2,13]. Sexual dimorphism in rodents appears to be regulated by estrogen secretion in adult females and by neonatal and adult androgen secretion in males. In adulthood, the male characteristic liver metabolism is dependent on continuous androgen exposure. Neonatal exposure to testosterone imprints the male process of neuroendocrine control over pulsatile pituitary GH secretion, which is first seen during puberty, when the adult pattern of GH secretion becomes evident, and continues through adulthood. If androgen re-programming does not occur, the feminine secretion pattern remains (continuous GH secretion). In postpubertal rats, the male blood pattern consists of high-amplitude pulses (approximately 200 ng/mL) spaced approximately 3–4 hours apart with no measurable trough levels. In contrast, the female pattern consists of continuous low-amplitude pulses (25–50 ng/mL), and GH is always present. The sexually dimorphic pattern of GH secretion is also seen in humans, although it is not as marked as it is in the rat. Interestingly, the depletion of liver-derived IGF-I in male mice causes the feminization of some GH-regulated sexually dimorphic liver function markers. The loss of the feedback effect exerted by IGF-I on the hypothalamic-pituitary system results in increased GH secretion and includes elevated baseline GH levels between the pulses, which resemble the female pattern of pituitary GH release.

## 3. The Cellular Regulation of GH Signaling

**Figure 2:**
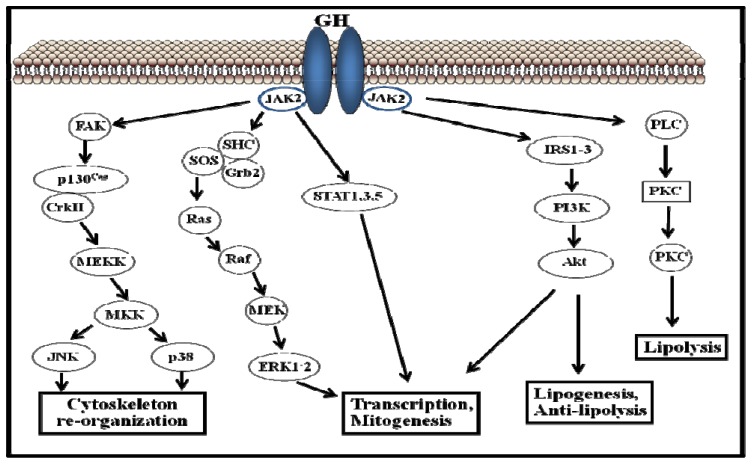
Schematic representation of growth hormone-activated signalling pathways.

The GHR belongs to the type I cytokine receptor, a family of receptors without intrinsic kinase activity [33]. Figure 2 shows the traditional view of the initiation of GH signaling: one molecule of GH binds two GHR monomers and induces their dimerization. Through trans-phosphorylation, GH binding to the GHR results in the activation of adjacent JAK2 molecules and cytoplasmic tyrosine kinases associated with the GHR. Activated JAK2 phosphorylates the GHR on tyrosine residues, which in turn recruits members of the STAT family of transcription factors. Of the various STAT proteins (STAT 1 to 6), STAT5b has been widely associated with GH biological actions; however, STAT1, 3, and 5a have also been shown to be recruited by the GHR. STAT5 phosphorylation by JAK2 results in their dissociation from the receptor, dimerization, and translocation to the nucleus, where STAT5 modulate the transcription of the target genes (e.g., IGF-I, ALS, SOCS2, SOCS3, CIS) [34,35]. The STATs represent one of five known pathways in GH-induced signaling; other pathways include the MAPK and PI3K (Figure 2).

The duration of GH-activated signals is a critical component of this hormone’s biological actions. Studies of the primary hepatocytes and several cell lines have shown that GH-induced JAK2-STAT5 activation is transient and that maximal activation is achieved within the first 30 min of stimulation, followed by a period of inactivation [36]. This process is clearly illustrated in the case of hepatic GH actions, in which the signal duration regulates gender differences in the liver gene expression [37,38]. As mentioned above, the male pattern of GH secretion in rats is episodic and peaks every 3–4 hours, with no measurable trough levels. This inactive period is characterized by an inability to achieve maximal JAK2-STAT5 activation by GH in the following 3 hours unless the GH is withdrawn from the media. Consequently, the intracellular activation of STAT5 is also episodic, and periods of low GH circulating levels are required to achieve the maximal activation of STAT5. Female rats exhibit a more continuous GH secretion pattern with higher basal levels and smaller, irregular intermittent peaks that show reduced STAT5b activation when compared with male rats. The differences in STAT5b activation are responsible for several of the gender differences in the hepatic gene expression [39]. GHR cell surface levels are the primary determinant of GH responsiveness [40]. Transcriptional, translational, and posttranslational level factors can influence GHR synthesis, thereby regulating the cell sensitivity to the GH actions. These factors include the nutritional status, the endocrine context, the developmental stage, and estrogens [36]. The removal of cell surface GHRs by endocytosis is an early step in the termination of GH-dependent signaling. By modulating both GHR internalization and proteasomal degradation, GHR ubiquitination is a key control mechanism in the down-regulation of GH signaling. In addition to GHR down-regulation, other mechanisms are required to complete the inactivation of GH signaling. Because the activation of GH-dependent signaling pathways is critically based on the protein phosphorylation of tyrosine, serine or threonine residues, the obvious mechanism to deactivate this process is the action of protein phosphatases. Recently, several studies have identified the phosphatases that are involved in the specific inactivation of GHR signaling. The signal regulatory protein (SIRP)-α, which belongs to a family of ubiquitously expressed transmembrane glycoproteins, negatively regulates GH-activated signaling by inhibiting the phosphorylation of JAK2, STAT5b, STAT3, and ERK1-2 [41]. Finally, SOCS proteins [42] have been identified as key components of the negative regulators of the GHR-JAK-STAT signaling pathway. SOCS proteins have been shown to modify cytokine actions through a classic negative feedback loop. In general, SOCS protein levels are constitutively low; however, their expression is rapidly induced by the stimulation of different cytokines or by growth factors, such as GH. SOCS proteins bind the receptor/JAK complex and down-regulate the JAK-STAT signaling pathway. Particularly, the phenotype of SOCS2 null mice (SOCS2KO) identifies SOCS2 as a key physiological player in the negative regulation of GH signaling [43,44]. Other studies have demonstrated that SOCS2 is essential for the regulation of the GH actions that are indirectly related to somatic growth. For example, SOCS2 blocks the GH-dependent inhibition of neural stem cell differentiation. Consequently, SOCS2KO mice have fewer neurons in their developing cortexes, whereas SOCS2 overexpression results in increased neural differentiation. Recently, it has been demonstrated that SOCS2 inhibits intestinal epithelial [45] and prostate cell proliferation [46], which are induced by GH *in vivo*. Evidence also indicates that other hormones, e.g., insulin, xenobiotics, and steroid hormones (including estrogens), can induce SOCS expression [42]. Consequently, the regulation of SOCS protein expression provides a mechanism for crosstalk, and multiple factors can regulate the activity of specific cytokines. Particularly, SOCS2 may be a physiological mechanism by which estrogen can suppress GH-dependent JAK2 phosphorylation [15].

## 4. STAT5 Plays a Relevant Role in GH-Dependent Regulation of Body Growth and Composition, Liver Metabolism and Gender-Dependent Dimorphism

GH exerts its physiological influence through transcriptional regulation and acute changes in the catalytic activity of several enzymes [4,8,47,48,49]. The global expression microarray analysis of GH actions in the liver clearly indicates that most of the known physiological effects of GH can be explained through its effect on the transcription of specific genes [6,34,39,50]. To this end, GH is known to activate a network of transcription factors in the liver, including nuclear receptors/transcription factors, such as HNF (4α, 6, 3β, PPARβ, CAR, FXR, SHP, SREBP, CRBP, C/EBPβ, and STAT5b. Based on the gene ontology analysis of liver transcript profiles from the targeted disruption/mutation of GHR-signaling pathway components (or GHR itself) and GH administration to GH-deficient mice and rats, the main metabolic process affected by GH status is energy/fuel metabolism, particularly lipid/fat metabolism; the metabolism of carbohydrates, proteins, steroids, and drugs is also strongly influenced. Combined with the clinical studies of GH-insensitive mutants, these animal findings have revealed that the transcription factor STAT5b is a key GH signaling intermediary for the regulation of postnatal growth, lipid metabolism, and the sexual dimorphism of hepatic gene expression. In addition, many transcripts are regulated independently of STAT5b, presumably as a result of the GHR-dependent activation of the ERK, Src, and PI3K signaling pathways.

### 4.1. Body Growth

GH is predominantly linked with linear growth during childhood. The liver is a major GH target tissue and the principal source of circulating IGF-I. The GH-dependent transcription of IGF-I is regulated by STAT5 binding sites in the IGF-I gene [51]. Thus, both IGF-I and its transcriptional regulator STAT5 have key roles in mediating the actions of GH in body growth [28,51]. Importantly, intermittent (male pattern) GH administration in rodents is a more potent stimulus of body growth rate, IGF-I expression, and STAT5b nuclear translocation in the liver than continuous (female pattern) GH administration. This difference supports the notion that the larger body growth in male rodents compared to female rodents could be caused by more effective stimulation of IGF-I and STAT5b mediated transcription. IGF-I proteins are also induced by GH in many tissues, and the local induction of IGF-I in chondrocytes plays an important role in longitudinal growth. However, GH is more effective than IGF-I because GH exerts additional growth-promoting actions independently of IGF-I [52].

Global disruption of STAT5b in mice causes the loss of sexual dimorphic growth characteristics, which reduces the affected males to the size of females; the female mice appeared unaffected [50]. Parallel observations were made with serum IGF-I concentrations, which were reduced by 30–50% in the affected male mice but not in the females. However, the combined disruption of STAT5a/b significantly reduced weight gain in the female mice and suppressed their body growth more than in the STAT5b null mice alone; the results approached the levels that were observed in either the GH- or GHR-deficient mice [35]. These studies demonstrated that STAT5b is important for male-specific body growth, whereas STAT5a regulates body growth in both sexes. Experiments on SOCS2KO mice also support the notion that STAT5b is critical for GH-regulated growth in mammals [42]. Importantly, SOCS2KO mice have enhanced growth, whereas combined STAT5bKO and SOCS2KO mice do not, demonstrating the necessity of STAT5b for the excess body growth observed in the SOCS2KO mice. In addition to the endocrine actions, the paracrine involvement of STAT5a/b in the interaction between GH and muscle is evidenced by the loss of muscle and mass in the IGF-I transcripts, which is observed with the muscle-specific deletion of STAT5a/b [53]. As mentioned above, the growth of female STAT5bKO mice is normal, whereas postnatal growth in GHR-deleted female mice is profoundly retarded. These data suggest that in addition to STAT5b, other transcription factors are related to growth. This relationship is exemplified by the glucocorticoid receptor (GR), which is a critical co-activator of STAT5b in the liver [54]. Importantly, the STAT5b and GR co-regulated transcripts were preferentially enriched in the functional groups related to growth and maturation (*i.e*., IGF-I). Moreover, both direct and indirect interactions between ERs and STAT5 [55] should be added to the list of mechanisms that are regulated by the nuclear receptors that modulate GH-dependent transcription.

### 4.2. Metabolism

GH exerts important metabolic actions throughout life. The metabolic effects of GH predominantly involve the stimulation of lipolysis in the adipose tissue, which results in an increased flux of free fatty acids (FFAs) into the circulation. In the muscle and liver, GH stimulates triglyceride (TG) uptake by enhancing the expression of lipoprotein lipase (LPL), and subsequent TG storage. The effects of GH on carbohydrate metabolism are more complicated and may be mediated indirectly via the antagonism of insulin action. Furthermore, GH has a net anabolic effect on protein metabolism because it stimulates protein synthesis while repressing proteolysis. GH has anabolic effects and increases muscle size in GH-deficient individuals [3,4,5].

The mechanisms of GH actions on lipid metabolism are complex and involve transcriptional and acute changes in catalytic enzyme activities [4,8,47,48]. It is well established that human GH is a lipolytic hormone. The long-term administration of GH includes a decrease in fat deposition and an increase in fat mobilization, thereby increasing circulating FFA and glycerol levels. GH reduces fat mass, particularly in individuals who have accumulated excess fat during periods of GH deficiency (GHD). Obesity is clinically evident in GHD patients, and a decline in GH levels correlates with age-related obesity. The lack of GH or GH signaling induces early obesity in mice [56,57]. Furthermore, GHD in adulthood causes a syndrome that is characterized by increased visceral adiposity, decreased muscle mass, metabolic disturbances, and increased mortality associated with cancer or vascular complications. This syndrome closely resembles metabolic syndrome and can be ameliorated by GH replacement [3,4]. Interestingly, the GH treatment of both healthy and GHD individuals decreases whole-body carbohydrate oxidation and concomitantly increases whole-body lipid oxidation. This process opens the possibility that the GH-induced increases in FFA efflux from adipose tissue could, via the provision of substrates for gluconeogenesis, abrogate the need for amino acids and proteolysis. The increased expression of β3-adrenergic receptor in adipocytes followed by the activation of hormone-sensitive lipase (HSL) is one of the GH mechanisms that lead to lipolytic effects. Additional effects include the uncoupling of the electron transport chain, which enhances mitochondrial heat generation at the expense of energy production from ATP. In the muscle and liver, GH stimulates the uptake and subsequent storage of TG by enhancing lipoprotein lipase (LPL) expression, contrary to the effect of GH on adipose tissue. GH stimulates TG uptake in the skeletal muscle primarily by increasing LPL expression, thereby promoting lipid utilization. However, several factors, such as nutrition, exercise, and sex steroid hormone status, could modify the GH-induced TG storage and lipid oxidation in the skeletal muscle. In the liver, GH treatment can induce a state of TG storage. Three possible mechanisms may be involved in this process: (a) the inhibition of intrahepatic TG (IHTG) lipolysis, (b) the inhibition of lipid oxidation, and (c) enhanced lipogenesis. There are data supporting all three hypotheses. In bGH-transgenic mice, there is a significantly reduced expression of hepatic HSL, suggesting that GH inhibits the lipolysis of IHTG. In addition, studies on bGH-transgenic, GHRKO, PPARαKO, and GH-treated rats (hypophysectomized or hypothyroid) have revealed that GH serves to down-regulate the genes involved in lipid oxidation (e.g., PPAR-α, acyl CoA oxidase, and CPT-1) and increases the expression of the genes promoting lipid synthesis (acetyl CoA carboxylase) in the liver [17,58,59,60]. Interestingly, the deletion of the STAT5 gene in the liver resulted in hepatic steatosis and increased phosphorylation of STAT1 and STAT3 under basal and GH-induced conditions, suggesting that GH may stimulate IHTG storage in a STAT5-independent manner. However, the deletion of the hepatic GHR gene in mice also resulted in hepatic steatosis because of enhanced lipogenesis and reduced TG secretion from the liver. However, these effects cannot be completely attributed to GH action on the liver because these mice had decreased levels of circulating IGF-I and hyperinsulinemia [17,59]. GHR-JAK2-STAT5 signaling deficiency has also been studied by the mutagenesis of GHR in mice, a model that causes severe obesity in mature mice in proportion to the loss of STAT5b activity [4]. Collectively, these experiments have shown that STAT5 regulates several key enzymes or genes that are otherwise involved in lipid and energy balance. Genetically modified animals and microarray analyses have provided new insights into the long-known anti-adiposity actions of GH and highlighted a key role for STAT5 in these actions. This role is supported by the original findings that STAT5b-deleted male mice become obese in later life [50] and that STAT5b deletion in a mature human was associated with obesity [61]. Based on altered transcript expression, several processes have been implicated. For example, the up-regulation of some lipogenic genes (e.g., CD36, PPARγ, PGC1α/β, FAS, SCD1, LPL, and VLDLR) may contribute to increased hepatic steatosis and adiposity in deficient GHR-JAK2-STAT5 signaling models, whereas the expression of antilipogenic genes, such as FGF21 and INSIG2, are decreased. The anti-obesity actions of GH are enhanced by the pulsatility of GH secretion, which is evident in males because pulsatile STAT5 activation, as mentioned above, is important for sexual dimorphism in hepatic gene expression (including IGF-1). Despite normal plasma FFA and minimal adiposity, the absent GHR activation could lead to hepatic steatosis because the activated STAT5 prevents this pathology [3].

### 4.3. Insulin Sensitivity

The effects of GH on both glucose and lipid metabolism are key components in the GH-dependent induction of insulin resistance. In the liver, GH has a stimulatory effect on glucose production, which may be a result of its antagonism of insulin action leading to hepatic/systemic insulin resistance. GH increases glucose production by increasing glycogenolysis; however, GH has either a stimulatory effect or no effect on gluconeogenesis. Moreover, over-expressing the human GH gene in rats increases the basal hepatic glucose uptake and glycogen content [62]. In contrast, the GHD (Ames) and GHRKO mice have improved insulin sensitivity and an up-regulation of hepatic insulin signaling, suggesting that GH antagonizes insulin signaling in the liver [63]. As mentioned above, GH-induced insulin resistance may develop from the increased FFA mobilization in the adipose tissue, which can affect liver insulin sensitivity and lead to insulin resistance and the up-regulation of PEPCK and G6Pase. However, the LID (IGF-I-specific liver deficient) mice show a 75% reduction in circulating IGF-I levels and a 3- to 4-fold increase in the circulating GH level and insulin resistance without a significant increase in the circulating FFA levels. Paradoxically, while crossing LID mice with GH transgenic mice, the serum FFA levels significantly increased, and there was an improvement in insulin sensitivity during a hyperinsulinemic-euglycemic clamp due to higher hepatic, adipose tissue and skeletal muscle glucose uptake [64]. This result suggests that in addition to FFA, other factors may also contribute to GH-induced insulin resistance. One candidate is the SOCS family of proteins, whose expression is induced by GH [42]. Another mechanism by which GH may induce insulin resistance is by increasing the expression of p85, a regulatory subunit of PI3K [3]. Finally, given the large homologies between the insulin and IGF-I systems, it is not surprising that IGF-I profoundly affects carbohydrate metabolism. Alternatively, IGF-I may enhance insulin sensitivity by suppressing GH release via negative feedback. Therefore, the activation of IGF-I signaling adds more complexity to the understanding of the molecular mechanisms involved in GH-induced insulin resistance *in vivo*.

### 4.4. Gender Dimorphism in the Liver

Sex-dependent gene expression and GH regulation characterizes several of the hepatic gene families involved in endobiotic and xenobiotic metabolism and relevant metabolic functions (e.g., lipid metabolism); 20–30% of all hepatic genes in rodents have a sex-specific expression pattern [39,47,48,49,50,65]. Most of these hepatic sex differences are explained by the female-specific secretion of GH through the induction of female-predominant transcripts and the suppression of male-predominant transcripts. STAT5b is a key player in this scenario. Results from experiments with STAT5b null mice have shown that STAT5b is responsible for the masculinization of the male liver. STAT5b binding sites have been found in the promoters of several sex-differentiated CYP rat genes (e.g., Cyp2c12, Cyp2c11, Cyp2a2). Conversely, other transcription factors (e.g., HNF6 and HNF3b) are more efficiently activated in the female liver or by the continuous GH secretion pattern. Sex differences are found in the hepatic genes involved in endobiotic and xenobiotic metabolism as well as in GH-regulated lipid metabolism. HNF4 and HNF3b are relevant transcription factors for regulating the genes involved in glucose and lipid metabolism [66,67], and they likely also contribute to sexual dimorphism. The continuous administration of GH has been shown to increase the hepatic expression of transcription factor SREBP-1c and its downstream target genes [68], as well as hepatic TG synthesis and VLDL secretion [69]. As mentioned above, GH actions in the liver lead to increased lipogenesis (*i.e*., SREBP1c induction) and decreased lipid oxidation (*i.e*., inhibition of PPARα) and promote anabolic growth in peripheral tissues (e.g., muscle and bone) [47,48,49]. In contrast, estrogens can cause the opposite effect (in comparison with GH) on hepatic lipid metabolism and insulin sensitivity, which represents a relevant point of regulatory interactions between estrogens and GH (see below).

## 5. The Liver Is a Target for Estrogen

The liver is a direct target of estrogens because it expresses ERα [12], which is connected with liver development [20], the regulation of hepatic metabolic pathways [11,25], growth [21], protection from drug-induced toxicity [22], hepatocarcinogenesis [23], fertility [24], lipid metabolism, and insulin sensitivity [11,25]. In addition, as mentioned above, estrogens can modulate the effects of GH on the liver by acting centrally, regulating pituitary GH secretion, and peripherally modulating GH signaling. Therefore, the liver represents a site where critical interactions can be developed between estrogens and GH.

### 5.1. Estrogen Receptor Signaling

Estrogens exert their physiological effects through two ER subtypes, ERα and ERβ, which belong to the nuclear receptor family of ligand-activated transcription factors [12]. Structurally, ERs share a common framework with the other members of the nuclear receptor family. The *N*-terminal A/B domain is the most variable region, with less that 20% amino acid identity between the two ERs, and confers specific subtype actions on targeted genes. This region harbors the activation function-1 (AF-1), which is ligand independent, and demonstrates promoter and cell-specific activity. The centrally located C domain harbors the DNA binding domain (DBD), which is involved in DNA binding and receptor dimerization. This domain is highly conserved between ERα and ERβ with 95% amino acid identity. The D domain is referred to as the hinge domain and shows low conservation between ERα and ERβ (30%). This domain has been shown to contain a nuclear localization signal. The *C*-terminal E domain is the ligand-binding domain (LBD), and the two subtypes display 59% conservation in this region. The LBD contains a hormone-dependent activation function (AF-2) and is responsible for ligand binding and receptor dimerization. The F domain has less than 20% amino acid identity between the two ER subtypes, and the functions of this domain remain undefined. Full transcriptional activity of the ERs is mediated through a synergistic action between the two activation domains: AF-1 and AF-2. Both ERα and ERβ contain a potent AF-2 function; however, unlike ERα, ERβ appears to have a weaker corresponding AF-1 function and depends more on the ligand-dependent AF-2 for its transcriptional activation function. The activities of the two ER subtypes are controlled by the binding of the endogenous hormone E2 or by synthetic non-hormonal compounds in the LBD. This binding triggers several events, such as overall conformational changes of the ERs, receptor dimerization and DNA binding to specific estrogen response elements, and interaction with coregulators (chromatin remodelers, coactivators, and corepressors), which are essential effectors in the biological activities of ligand-activated ERs. Each class of ER ligands induces a unique ER conformation that promotes specific coregulator protein interactions and associations between the ER *N*- and *C*-terminal transcription activation functions, AF-1 and AF-2, respectively [70]. E2, a nonselective agonist, has a similar affinity (Kd = 0.05–0.1 nM) for ERα and ER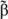 ER-subtype-selective agonists have been developed; PPT and DPN are ERα- and ERβ-selective agonists, respectively. In addition, the ERs bind a wide range of synthetic compounds with strikingly diverse structures, including selective estrogen receptor modulators (SERMs) (e.g., raloxifene). The SERMs are synthetic ER ligands that display tissue-selective pharmacology; as anti-estrogens (antagonists), they oppose the action of estrogens in certain tissues while mimicking the action of endogenous estrogens (agonists) in other tissues [70]. Environmental contaminants (e.g., polycyclic aromatic hydrocarbons, phthalates, pesticides), a class of estrogens termed xenoestrogens, and phytoestrogens also have estrogenic actions. Although their affinity for ERs is mostly 100 to 10,000 times lower than that of E2 [71], it is not questionable whether xeno- and phytoestrogens are biologically relevant in humans and farm animals. The tissue-selective expression of ERs can also determine estrogen physiology. ERα is mainly expressed in reproductive tissues, the kidney, bones, white adipose tissue, and the liver, while ERβ is expressed in the ovary, the prostate, the lungs, the gastrointestinal tract, the bladder, hematopoietic cells, and the central nervous system. Therefore, specific therapeutic actions of estrogens on tissues (e.g., the liver) may be obtained through selective ERα agonists (e.g., PPT) [72].

**Figure 3:**
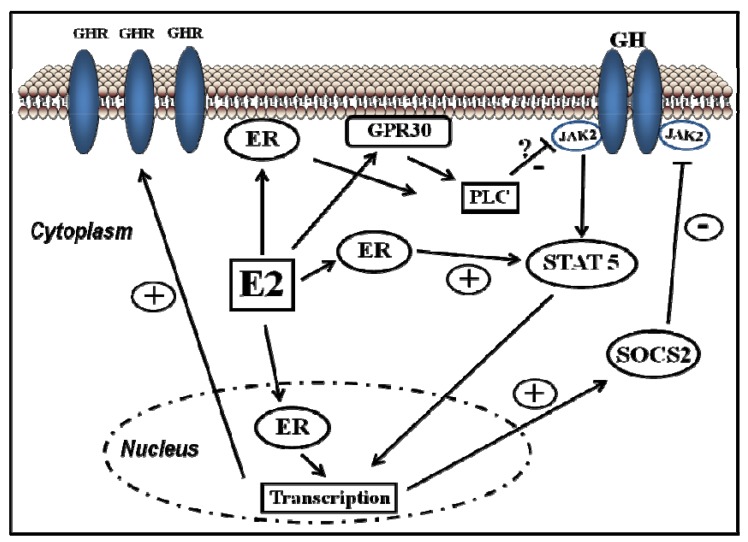
Signalling pathways activated by E2 and its crosstalk with growth hormone.

Classical estrogen signaling occurs through the direct binding of ER dimers to estrogen-responsive elements in the regulatory regions of estrogen-targeted genes followed by the activation of the transcriptional machinery at the transcription start site [12]. Estrogen also modulates gene expression by a second mechanism in which ERs interact with other transcription factors (e.g., STAT5) through a process referred to as transcription factor crosstalk. Estrogen may also elicit effects through non-genomic mechanisms, which involve the activation of downstream kinases pathways, such as PKA, PKC, and MAPK, via membrane-localized ERs. An orphan G protein-coupled receptor (GPR)-30 in the cell membrane has also been reported to mediate non-genomic and rapid estrogen signaling. In summary, the mechanisms involved in ER signaling are influenced by cell phenotype, the target gene, and the activity or crosstalk with other signaling networks. Figure 3 shows how E2 can interact with the GHR-JAK2-STAT5 signaling pathway. E2 can modulate GH actions through changes in GHR expression or through crosstalk with the GH-activated JAK2-STAT5 signaling pathway. The ERα-dependent induction of SOCS-2 followed by the inhibition of JAK2-STAT5 signaling is a relevant mechanism that could explain how estrogens directly inhibit GH-regulated actions in the liver [15,16]. We have observed that the long-term administration of physiological doses of E2 to GH-deficient male rats (hypothyroid) can induce the mRNA expression of SOCS-2 in the liver [16]. Hypothetically, other members of the STAT family of negative regulators may contribute to estrogen interaction by GH signaling in the liver [15]. The E2 activation of ERα or ERβ followed by direct interaction between ERs and STAT5 may also regulate STAT5-dependent transcriptional activity. Paradoxically, estrogens can also activate STAT5 signaling in a pituitary manner and even in a JAK2-independent manner [55,73,74,75].

### 5.2. E2 Modulates the GH Promoting of Skeletal Growth

It is well known that **s**ex steroids and GH interact closely to regulate pubertal growth [13]. In addition to the well-established role of the GH-IGF-I axis, estrogens are also known to play an essential role in the pubertal growth spurt and bone mineral accrual. Key findings in the context of estrogen resistance or deficiency include low bone mineral density and the failure to establish peak bone mass. Estrogen-deficient men experience no pubertal growth spurts and have a sustained linear growth without epiphyseal fusion. Estrogens induce the closure of the epiphyseal growth plate during late puberty, thereby limiting longitudinal growth and final bone size. During early puberty, estrogens stimulate longitudinal growth, a stimulatory effect that is considered to primarily reflect interaction with the GH-IGF-I axis. Estrogens stimulate GH secretion and the GH-induced hepatic synthesis of IGF-I [13]. Experiments with ER null mice have shown that ERα mediates important estrogen effects in the skeleton during growth and maturation [21]. Similar phenotypes can be found for aromatase-deficient rats, which cannot produce estrogens. Reports of natural mutations in the ERs and the aromatase gene in men, along with evidence from estrogen-resistant male mice and the administration of aromatase inhibitors in male rats, have also called attention to the physiological role of estrogens in skeletal growth. In addition to their interactions with the GH-IGF-I axis, estrogens can regulate pubertal skeletal growth and bone mineral acquisition independently of GH or GHR [76], suggesting that estrogens could rescue pubertal growth during GH resistance through a novel mechanism of independent GHR stimulation of IGF-I production in the liver (e.g., after activation through phosphorylation, STAT5 can stimulate IGF-I transcription). In contrast to physiological levels, the administration of pharmacological doses of estrogens results in the drastic reduction of circulating IGF-I, which most likely reflects the inhibitory effect of estrogens on the GH-JAK2-STAT5 signaling pathway in the liver (see below).

### 5.3. Gender Dimorphism in the Liver Is Regulated by the Pattern of GH Secretion and Sex Steroids

Genome-wide screens of gene expression have shown that the GH- and sex-dependent regulation of hepatic gene expression are not confined to steroid or drug metabolism, and a number of other hepatic genes have been found to be up- and/or down-regulated by the different patterns of GH or sex-steroid exposure [8,39,47,48,49,50,65]. GH- and sex-dependent hepatic transcripts encoding plasma proteins, enzymes, transcription factors and receptors involved in the metabolism of proteins, carbohydrates, lipids, or signaling regulation have been identified. There is a consensus that the response to different sex GH patterns is the major cause of gender dimorphism in the liver. However, it is also likely that factors other than the sexually dimorphic pattern of GH secretion are behind some sex differences in rat livers. Potential mechanisms that could contribute to “liver sexuality” are the pituitary-independent effects of estrogens through an interaction with ERα or the GH-JAK2-STAT5 signaling pathway in the liver.

### 5.4. E2 Regulates Lipid Metabolism and Insulin Sensitivity: Potential Crosstalk with GH

**Figure 4:**
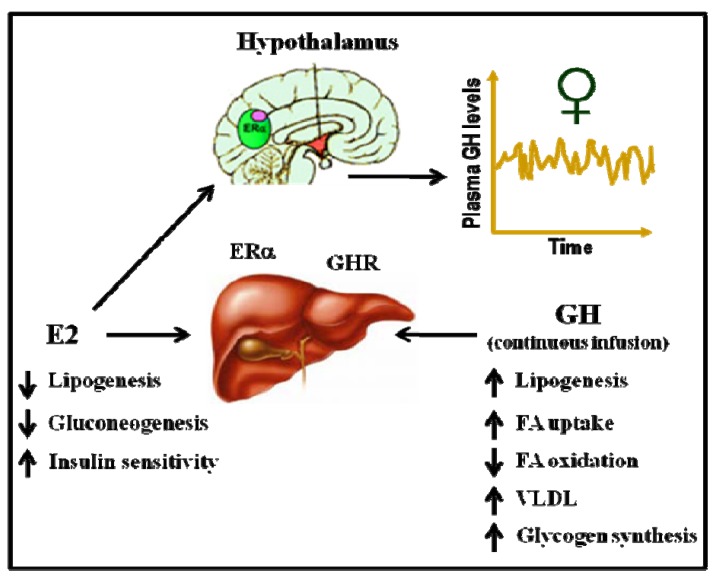
Physiological control of hepatic lipid metabolism by E2 and GH.

Acting on both ERα and ERβ, estrogens are recognized as important regulators of glucose homeostasis and lipid metabolism [75]. Several studies have shown that ERα controls inflammation, lipid, glucose, protein, and cholesterol homeostasis in the liver, leading to the conclusion that E2 via ERα is antidiabetogenic. In contrast, ERβ might be diabetogenic. Both male and female ERαKO mice develop insulin resistance and impaired glucose tolerance, similar to humans who lack ERα or aromatase. ERα mainly mediates the beneficial metabolic effects of estrogens, such as anti-lipogenesis, improved insulin sensitivity and glucose tolerance, and reduced body weight/fat mass. In contrast, ERβ activation appears to be detrimental for the maintenance of regular glucose and lipid homeostasis. The insulin resistance in ERαKO mice is largely localized to the liver, including increased lipid content and hepatic glucose production. Interestingly, the expression of liver lipogenic genes can be decreased after the administration of E2 to diabetic Ob/Ob or female mice fed high-fat diets. Similarly, the aromatase knockout mouse, which cannot produce E2, has increased intra-abdominal adiposity and develops steatosis and an impairment of lipid oxidation in the liver. As mentioned above, GH-GHR-JAK2-STAT5 deficiency in adults causes adiposity and hepatic steatosis. Therefore, E2 and GH regulate a common cellular network related to the physiological control of lipid metabolism (Figure 4). In our lab, we have shown that the subcutaneous administration of near-physiological doses of E2 to male rats with GH deficiencies (hypothyroid rats) dramatically influenced the hepatic transcriptional response to pulsatile GH administration (male pattern). Particularly, the expression of several genes related to the endocrine, metabolic, and sex-differentiated functions of GH were drastically inhibited by E2 [77].

## 6. The Modulation of GH Actions by Estrogens Is Clinically Relevant

Multiple regulatory interactions between estrogen and GH can be achieved in the liver. Observational studies in children have reported that puberty is significantly associated with increases in mean sex steroids, GH and IGF-I concentrations and the IGF-I response to an injection of GH. These observations suggest that the endogenous gonadal steroid milieu increases GH sensitivity in girls and boys during puberty. However, whether exogenous estrogen alters GH sensitivity during childhood must still be clarified. E2 can reduce the level of circulating IGF-I when used to prime GH stimulation tests in prepubertal children or when taken as an oral contraceptive or as hormone replacement therapy in menopausal or hypopituitary women. In adulthood, exposition to estrogens is associated with GH resistance. It is well accepted that oral estrogens can impair the metabolic and endocrine function of the liver in adulthood. Studies in women indicate that oral estrogens, particularly higher doses, impair the IGF-I response to GH [78,79,80]. GH (but not IGF-I) levels are higher in young women than in age-matched men. In GH-deficient males (compared to female patients), GH treatment induces a greater increase in lean mass and a decrease in fat mass or a greater increase in the indices of bone turnover and bone mass. IGF-I levels are lower in GH-deficient women, and the IGF-I increase in response to GH treatment is approximately half that of their male counterparts, which results in women requiring a higher replacement dose of GH than men. Studies of GH-deficient and postmenopausal women provide compelling evidence that estrogen levels achieved in the portal circulation after ingestion of therapeutic doses of oral estrogen impair GH-regulated liver functions. The oral administration of therapeutic doses of estrogen to hypopituitary patients can inhibit the endocrine and metabolic effects of GH; circulating IGF-I levels, lipid oxidation, and protein synthesis are suppressed, with a reciprocal elevation in carbohydrate oxidation [81]. The oral administration of estrogen led to a significant increase in fat mass and a loss of lean body mass compared to that observed during transdermal estrogen therapy. Interestingly, the effects on fat oxidation and IGF-I induced by the oral route of estrogen administration contrast the effects of GH and are consistent with an antagonistic effect on GH actions. In summary, therapeutic doses of estrogen may affect the endocrine and metabolic actions of GH in the liver and are somehow different from the physiological doses of E2. Estrogen inhibits hepatic IGF-I production in a concentration-dependent manner regardless whether this inhibition is achieved through the portal or systemic circulation [82,83,84]. As mentioned above, estrogen can inhibit the effects of GH in the liver by inducing the negative regulators of GH signaling [15,85].

## 7. Conclusions

Estrogen interactions with GH can be executed indirectly at the level of pituitary GH secretion and directly at the cellular level. The impact of estrogens on GH-regulated endocrine (e.g., IGF-I), metabolic (e.g., lipid metabolism, insulin sensitivity), and sex-differentiated (e.g., lipid, endobiotic and xenobiotic metabolisms) functions in the liver are physiologically and therapeutically relevant. The detrimental impact of oral estrogens on the metabolic actions of GH is clinically relevant. Furthermore, SERMs and phytoestrogens are gaining widespread use. The endocrine and metabolic consequences of long-term exposition to novel estrogen-related compounds are still largely unknown. These complex interactions deserve further research because they can potentially impact human health.
